# Items

**Published:** 1898-08

**Authors:** 


### ITEMS.

We invite the attention of physicians to St. Mary's Maternity and Infant Asylum on Edward street, this city. It is one of the oldest institutions of the kind in the country, having been founded in 1854, and affords advantages for maternity cases unexceled anywhere. Physicians sending cases to it, can be assured of the utmost secrecy when that is desired. They can also attend their own private patients while in the institution, if it is their wish to do so ; otherwise skilled obstetric service will be furnished. Both public and private cases receive every attention and comfort possible and the nursing is in every way up to date. Homes are provided for infants after confinement when desirable. See the prospectus of the institution among our advertisements, page xxvi.

---

The Imperial Granum Food Company, New Haven, has issued a chart for recording the vital signs and other information relating to the baths given in the treatment of fever cases. It is very complete and will be found especially useful in typhoid fever. Write to the company for their new nursing world fever chart.

---

The Niagara Pharmacal Co. has just completed extensive laboratories and showrooms at the corner of South Division and Ellicott streets (opposite the new postoffice), Buffalo. It not only manufactures a full line of fluid extracts, tablets and other pharmaceuticals, but also carries a complete stock of the products of every other prominent laboratory in the country. The preliminary catalogue issued by this company indicates that it is in position to furnish physicians and surgeons with every requisite in the practice of either branch of the profession.

